# Teriparatide Replacement Therapy for Hypoparathyroidism During Treatment With Lenvatinib for Advanced Thyroid Cancer: A Case Report

**DOI:** 10.3389/fendo.2018.00244

**Published:** 2018-05-17

**Authors:** Tommaso Porcelli, Francesca Sessa, Angela Caputo, Christian Catalini, Domenico Salvatore

**Affiliations:** Department of Clinical Medicine and Surgery, University of Naples Federico II, Naples, Italy

**Keywords:** metastatic thyroid cancer, tyrosine kinase inhibitors, targeted therapy, lenvatinib, postsurgical hypoparathyroidism, recombinant human parathyroid hormone (1–34), teriparatide

## Abstract

Thyroid cancer metastasizes in 4% of cases. Approximately two-thirds of these patients are refractory to radioactive iodine-131 (RAI) therapy and have a poor 10-year survival prognosis. Treatment with tyrosine kinase inhibitors (TKIs) may be administered in selected RAI-refractory patients. However, these agents are often associated with adverse events, including vomiting. We report the case of a patient affected by RAI-refractory thyroid cancer with lung and intracranial metastases undergoing treatment with the antiangiogenic TKI lenvatinib, and with teriparatide replacement therapy for postsurgical hypoparathyroidism. Due to lenvatinib-related vomiting, which did not respond to therapy, conventional oral calcium supplementation failed to maintain normal serum calcium levels and the patient had repeated episodes of hypocalcemia. Subcutaneous teriparatide injections restored serum calcium levels, and thus lenvatinib therapy could be continued. This experience indicates that hormone replacement with teriparatide is a feasible option for cancer patients affected by hypoparathyroidism not treatable with oral calcium supplementation.

## Background

Patients affected by thyroid cancer have a 5-year survival rate of 98.2%. Distant metastases occur in approximately 4% of cases (1). Two-thirds of follicular cell-derived metastatic thyroid cancers do not respond adequately to radioactive iodine-131 (RAI) ablation therapy. In fact, they either fail to uptake iodine at the first ablation or become refractory to treatment. The prognosis for RAI-refractory (RAI-R) patients is poor, the 10-year overall survival rate being <10% (2, 3). The recommended treatment for patients with metastatic, rapidly progressive, symptomatic, and/or imminently threatening RAI-R cancer who do not respond to locoregional treatments is systemic therapy with tyrosine kinase inhibitors (TKIs) (4). Lenvatinib is a multitargeting antiangiogenic agent approved for progressive advanced RAI-R thyroid cancer (5). TKIs have class-related adverse events and vomiting is a common event in patients treated with antiangiogenic agents (6).

Here we report the first case of a patient affected by progressive metastatic thyroid cancer and postsurgical hypoparathyroidism, in whom TKI-induced vomiting impaired oral calcium intake thereby causing severe hypocalcemia. Teriparatide (recombinant human parathyroid hormone, rhPTH (1–34)) replacement therapy allowed restoration of circulating calcemic levels without discontinuation of oncological therapy.

## Case Report

A 66-year-old woman presented with a recent history of diplopia and right palpebral ptosis. She had undergone total thyroidectomy in another Institute in June 2015 due to the enlargement of a nodule in the right thyroid lobe within a longstanding goiter. Pathology showed a Hürthle cell carcinoma, 7 cm maximum diameter, with extensive vascular invasion that involved the perithyroidal soft tissue. Due to postsurgical hypoparathyroidism, the patient started substitutive therapy with oral calcium and activated vitamin D. In November 2015, she received a dose of 142.4 mCi RAI. Her stimulated thyroglobulin (Tg) level at that time was 8.9 ng/ml with undetectable thyroglobulin antibodies (Tg-Ab). Therapeutic whole body scan (RxWBS) revealed non-specific uptake in the left paratracheal region. Neck recurrence appeared a few months later and, in July 2016, the patient received a 214.6 mCi dose of RAI. RxWBS was negative, whereas SPECT/CT fusion images revealed RAI-refractory tumor tissue in the right supraclavicular region. Consequently, the patient underwent surgery in August 2016 and an infiltrative TTF1 and Tg-positive lesion, 3 cm maximum diameter, was removed. However, in November 2016, basal Tg increased to 126 ng/ml and an ^18^FDG PET/CT revealed disease persistence in the right neck and multiple bilateral lung micrometastases.

**Figure 1:**
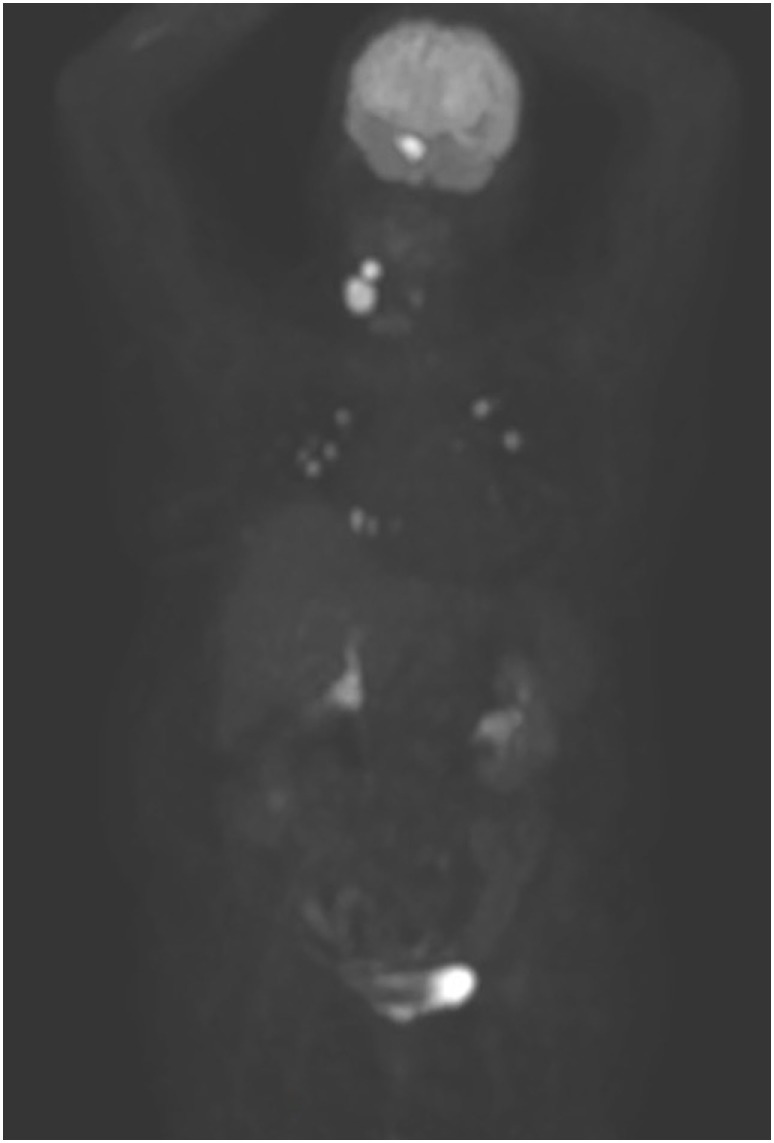
^18^FDG PET/CT performed before lenvatinib administration. Besides the numerous bilateral lung metastases, note the intense uptake by two locally recurrent lesions in the neck and by the cavernous sinus metastasis.

**Figure 2:**
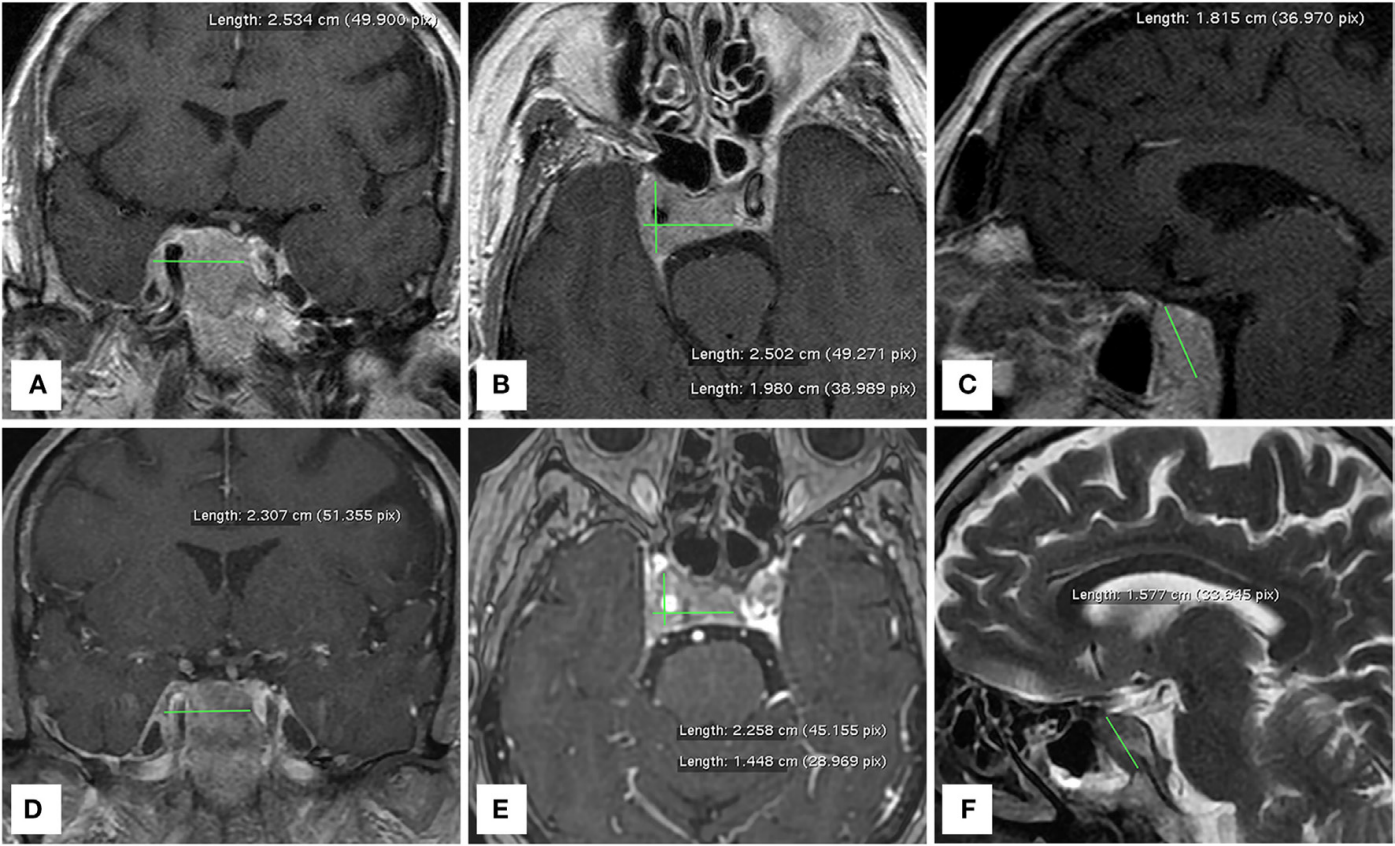
MRI brain scans performed before **(A–C)** and one month after **(D–F)** the administration of lenvatinib. The right cavernous sinus metastasis completely encapsulates a consistent portion of the intracranial tract of the right internal carotid artery.

**Figure 3:**
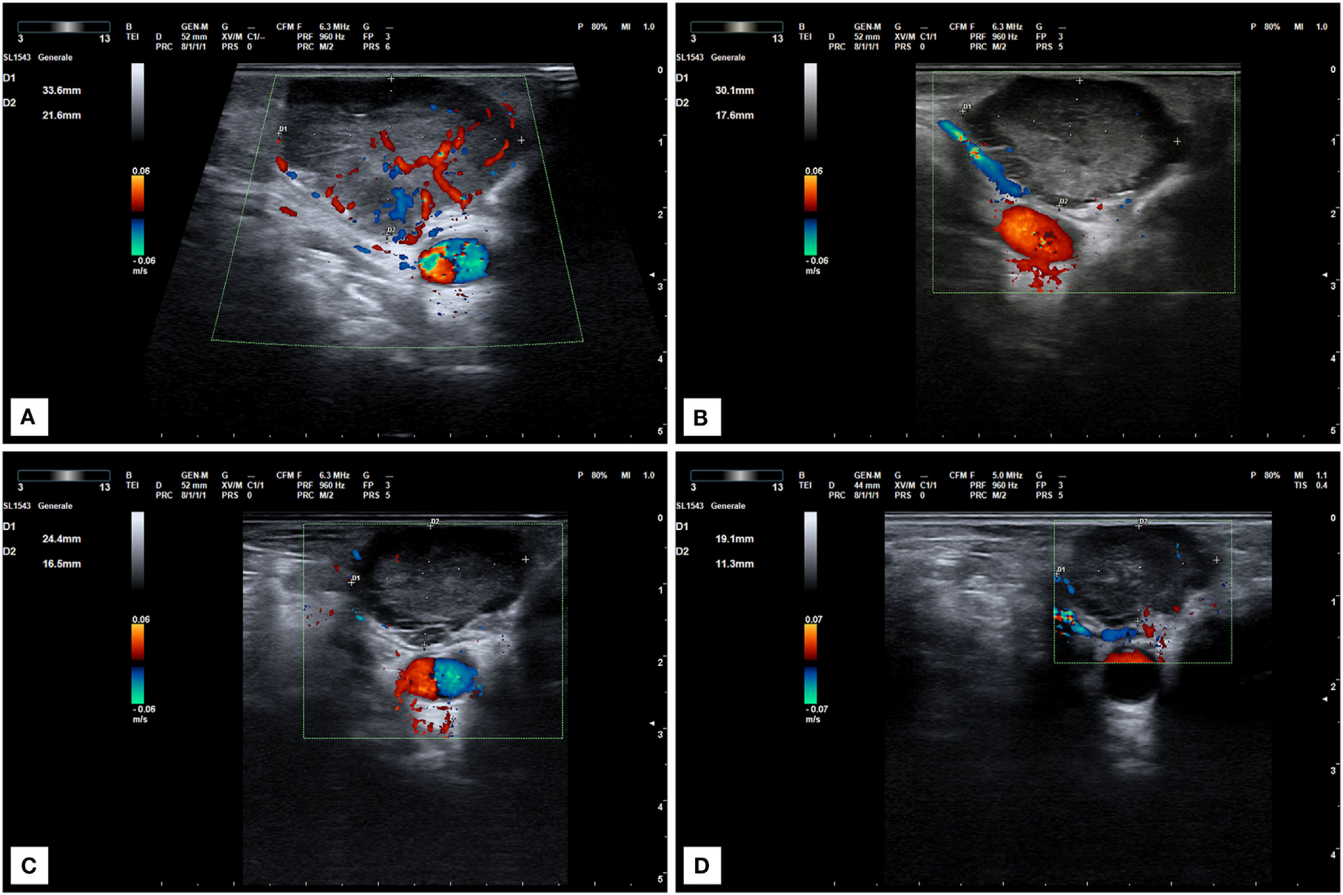
Ultrasound scan of a locally recurrent neck lesion in right supraclavicular region. **(A)** Imaging before lenvatinib start. **(B)** Ultrasound scan after 1 month of therapy at 10 mg daily. Tumor vascularization is significantly reduced. **(C,D)** Progressive tumor reduction after 2 **(C)** and 6 months **(D)** of treatment at the same dose.

At our first observation in May 2017, basal Tg was 500 ng/ml, Tg-Ab <20 U/ml, TSH 0.015 mU/l, and daily levothyroxine intake was 137.5 mcg. Diplopia and right palpebral ptosis had started about 1-month earlier. A subsequent ^18^FDG PET/CT scan showed intense glucose metabolism in the basisphenoid region (Figure 1) and a gadolinium-enhanced brain MRI scan revealed a mass in the right cavernous sinus measuring 2.5 cm × 2.0 cm × 1.8 cm, with a low signal in T2 and enhancement on post-contrast images, which was compatible with a metastasis. The lesion encapsulated a 2-cm segment of the right internal carotid artery, without causing luminal narrowing (Figures 2A–C). Given the patient’s non response to RAI treatment and the locally advanced and progressively increasing metastatic burden, lenvatinib was initiated in June 2017. Because the intracranial lesion encapsulated a considerable portion of the right internal carotid artery, we started with a low dose (10 mg daily) to avoid the risk of artery damage consequent to rapid tumor shrinkage. An MRI performed about 30 days later showed that the metastasis in the right cavernous sinus was significantly smaller and that the integrity of the internal carotid was preserved (Figures 2D–F). Diplopia and palpebral ptosis progressively regressed with treatment and the patient did not complain of any cerebral symptom. Basal Tg declined to 338.4 ng/ml without Tg-Ab, in line with a reduction in neck lesions volume (Figure 3).

Two weeks later, the patient had grade 2 vomiting and was treated with serotonin 5-HT3 receptor antagonists. However, vomiting worsened to grade 3, and lenvatinib was suspended for 1 week. Despite the transdermal administration of granisetron, grade 1 vomiting persisted and was exacerbated by the oral assumption of calcium that the patient needed to control the severe hypoparathyroidism. Notwithstanding the administration of high doses of calcitriol, the hypocalcemic episodes were frequent and the patient underwent intravenous calcium gluconate infusion in various hospital emergency departments. Because of vomiting after oral calcium intake, it was not possible to restore normal serum calcium levels and the patient became dependent on intravenous therapy.

Due to the patient’s poor clinical condition, we decided to admit her to our department and administered 20 µg teriparatide in daily subcutaneous injections as replacement therapy for hypoparathyroidism. Intravenous calcium infusion was progressively reduced and was suspended after 1 week, when serum calcium levels became stable at a dose of 20 µg teriparatide twice daily. No adverse event occurred. Subsequent controls showed normal serum calcium and phosphorus levels; no clinical signs of hypocalcemia were reported. At the last control in March 2018, basal Tg was 82 ng/ml (without Tg-Ab), serum calcium was 8.8 mg/dl, and phosphorus was 3.3 mg/dl.

## Discussion

This is the first report of the use of teriparatide as hormonal replacement therapy for hypoparathyroidism in a patient with metastatic thyroid cancer. Our patient was affected by rapidly progressive macroscopic refractory disease and was on systemic therapy with the antiangiogenic multikinase inhibitor lenvatinib. Patients affected by advanced thyroid cancer are prone to parathyroid damage and postsurgical hypothyroidism because they undergo aggressive neck surgery (7). Therapy with a systemic TKI is indicated in case of substantial disease progression not otherwise treatable (4). Vomiting is a common class-related adverse event of antiangiogenic TKIs (6). Since current treatment for hypoparathyroidism consists of activated vitamin D and calcium, both of which must be taken orally, intractable vomiting places patients at a high risk of hypocalcemia. Consequently, such patients require alternative therapeutic options.

After a lenvatinib-associated G3 adverse event, it is common practice to suspend treatment and resume it at a lower dosage (8). In our case, the patient was already on a low dose, namely, 10 mg daily. Therefore, after a 1-week drug-free interval, we associated 10 mg lenvatinib daily with transdermal granisetron. Vomiting was controlled but oral calcium assumption triggered intolerable vomiting. Therefore, conventional supplementation for hypoparathyroidism could not be used to correct the patient’s hypocalcemia. Continuity of therapy is crucial for patients undergoing treatment with a TKI (9). Thus, to treat hypoparathyroidism without interrupting lenvatinib therapy, we administered replacement therapy with subcutaneous teriparatide. In Italy, rhPTH (1–34) as substitutive therapy for severe chronic hypoparathyroidism became reimbursable by the National Health Service in June 2013. Inclusion criteria for this treatment are non response to oral activated vitamin D and recurrent severe episodes of hypocalcemia. Contraindications for treatment are previous external beam radiation therapy to the skeleton and bone malignancies or bone metastases (10).

Concerns about the carcinogenic potential of long-term rhPTH (1–34) treatment were raised consequent to evidence obtained in rats of increased osteosarcoma risk in relation to dose and treatment duration (11). However, clinical studies conducted over a period of 10 years did not find an increase in the incidence of osteosarcoma in patients taking teriparatide. Similarly, the risk of malignancies was not increased in patients on long-term replacement with rhPTH (1–34) (12–14). In our case, the benefit of normalization of serum calcium levels largely outweighed the potential risk related to treatment with teriparatide.

## Concluding Remarks

During anticancer treatment, vomiting unresponsive to therapy can be a major issue for hypoparathyroidal patients who are dependent on oral calcium intake. This case shows that replacement therapy with teriparatide can be considered for the treatment of cancer patients who do not have bone metastases and are intractable to oral calcium supplementation for postsurgical hypoparathyroidism.

## Ethics Statement

The patient provided written informed consent for research participation and for the publication of indirectly identifiable data.

## Author Contributions

TP, FS, AC, CC, and DS managed the case. TP wrote the manuscript. All the authors revised and approved the final manuscript and agreed to be accountable for the content of the work.

## Conflict of Interest Statement

The authors declare that this case report was carried out in the absence of any personal, professional, or financial relationships that could potentially be construed as a conflict of interest.
